# Biochemical and Functional Characterization of Glycoside Hydrolase Family 16 Genes in *Aedes aegypti* Larvae: Identification of the Major Digestive β-1,3-Glucanase

**DOI:** 10.3389/fphys.2019.00122

**Published:** 2019-02-28

**Authors:** Raquel Santos Souza, Maiara do Valle Faria Gama, Renata Schama, José Bento Pereira Lima, Hector Manuel Diaz-Albiter, Fernando Ariel Genta

**Affiliations:** ^1^Laboratory of Insect Biochemistry and Physiology, Oswaldo Cruz Institute, Oswaldo Cruz Foundation, Rio de Janeiro, Brazil; ^2^Laboratory of Systems and Computational Biology, Oswaldo Cruz Institute, Oswaldo Cruz Foundation, Rio de Janeiro, Brazil; ^3^Laboratory of Physiology and Control of Arthropod Vectors, Oswaldo Cruz Institute, Oswaldo Cruz Foundation, Rio de Janeiro, Brazil; ^4^Wellcome Centre for Molecular Parasitology, University of Glasgow, Glasgow, United Kingdom; ^5^National Institute of Science and Technology for Molecular Entomology, Rio de Janeiro, Brazil

**Keywords:** *Aedes aegypti*, β-1,3-glucanase, knock-down, digestion, immunity, Glycoside Hydrolase Family 16, GHF16

## Abstract

Insect β-1,3-glucanases belong to Glycoside Hydrolase Family 16 (GHF16) and are involved in digestion of detritus and plant hemicellulose. In this work, we investigated the role of GHF16 genes in *Aedes aegypti* larvae, due to their detritivore diet. *Aedes aegypti* genome has six genes belonging to GHF16 (Aae GH16.1 – Aae GH16.6), containing two to six exons. Sequence analysis suggests that five of these GHF16 sequences (Aae GH16.1, 2, 3, 5, and 6) contain the conserved catalytic residues of this family and correspond to glucanases. All genomes of Nematocera analyzed showed putative gene duplications corresponding to these sequences. Aae GH16.4 has no conserved catalytic residues and is probably a β-1,3-glucan binding protein involved in the activation of innate immune responses. Additionally, *Ae. aegypti* larvae contain significant β-1,3-glucanase activities in the head, gut and rest of body. These activities have optimum pH about 5–6 and molecular masses between 41 and 150 kDa. All GHF16 genes above showed different levels of expression in the larval head, gut or rest of the body. Knock-down of AeGH16.5 resulted in survival and pupation rates lower than controls (dsGFP and water treated). However, under stress conditions, severe mortalities were observed in AeGH16.1 and AeGH16.6 knocked-down larvae. Enzymatic assays of β-1,3-glucanase in AeGH16.5 silenced larvae exhibited lower activity in the gut and no change in the rest of the body. Chromatographic activity profiles from gut samples after GH16.5 silencing showed suppression of enzymatic activity, suggesting that this gene codes for the digestive larval β-1,3-glucanase of *Ae. aegypti*. This gene and enzyme are attractive targets for new control strategies, based on the impairment of normal gut physiology.

## Introduction

Culicides are important mosquitoes in the epidemiology of vector-borne diseases. They were associated with the spread of several infectious diseases since the beginning of the XX century (Ross, 1911; Brathwaite et al., 2012). *Ae. aegypti* is the main vector of Dengue, Urban Yellow Fever, Chikungunya, and Zika viruses in humans (Rodhain and Rosen, 1997; Jentes et al., 2011; Leparc-Goffart et al., 2014; Freitas et al., 2016). *Ae. aegypti* maintains a strong association with humans, breeding in virtually any container that holds enough water for larval/pupal development (Nelson et al., 1976). These containers are widely available in most developing countries, where water distribution and sanitary conditions are defective (Consoli and Lourenço-de-Oliveira, 1994). Due to a strong dietary preference for human blood, *Ae. aegypti* is capable of completing the entire life cycle within human dwellings (Edman et al., 1992). In this respect, *Ae. aegypti* is a vector of enormous medical importance and probably will continue to be for the next years to come (Weaver and Reisen, 2010).

The dengue virus has become the most important arboviral pathogen in recent years, due to its increasing incidence in the tropics and subtropics, as well as its high mortality and morbidity (Murray et al., 2013). It resembles malaria in geographic distribution and could be more meaningful regarding morbidity and economic impact (Gubler, 2002, 2012; Guzman et al., 2010; Bhatt et al., 2013). The WHO estimates dengue virus reaching 390 million people per year and 3.9 billion people are living at risk in 128 countries around the world (Brady et al., 2012; World Health Organization [WHO], 2017a). Yellow fever affects around 200,000 people a year, and Chikungunya virus during one outbreak infected more than 1.5 million people in India. Recently, the Zika virus infection has been recognized as a Public Health Emergency of International Concern, due to the related number of newborn infants with microcephaly and high incidence of Guillain-Barré syndrome cases (Vainio and Cutts, 1998; Mavalankar et al., 2008; World Health Organization [WHO], 2016, 2017b). These undesired outcomes increase the need for the creation of new methods to block the development of the mosquito (World Health Organization [WHO], 2013). Current strategies for facing these infectious diseases rely almost exclusively on vector control efforts (Maciel-de-Freitas et al., 2012; World Health Organization [WHO], 2012). There are no drug treatments, and the Dengue vaccine licensed in 2015 was not completely safe and efficient to protect against hospitalization due to dengue and severe dengue in all ages groups (World Health Organization [WHO], 2009, 2017a).

Most studies about *Ae. aegypti* have focused in the physiology of female adults and the knowledge about larval behavior is poor (Clements, 2000; Borovsky, 2003; Chen et al., 2008; Oviedo et al., 2008; Venancio et al., 2009; Douglas, 2014). The occurrence of *Ae. aegypti*-transmitted diseases is majorly determined by the presence of larval breeding sites (Kay, 1999). Thus, information on larval physiology and biochemistry may expand biological knowledge and result in new insights for vector control (Coutinho-Abreu et al., 2010).

In insects, digestion and absorption of its products occur mainly in the digestive tract (Terra and Ferreira, 2005). The gut is a major surface where exchanges with the external environment take place, being a strategic topic of investigation for the screening of targets for insect control. *Ae. aegypti* larvae have a detritivore feeding mode, filtering solid particles from liquid media and scraping organic material from surfaces. Several microorganisms such as bacteria, fungi, algae, protozoa, and rotifers have been found inside the gut of mosquito larvae (Walker et al., 1988; Merritt et al., 1990; Ho et al., 1992; Avissar et al., 1994; Muniaraj et al., 2012), but the mechanisms used by larvae for the breakdown of these nutritional sources remain unexplored.

β-1,3-glucans are ubiquitous polysaccharides. They are produced by many organisms such as algae, higher plants, and fungi. β-1,3-glucanases are enzymes capable of hydrolyzing β-1,3 bonds present in β-1,3-glucans. β-1,3-glucanases play an important role in the digestion of detritorous or grass-eating insects (Terra and Ferreira, 1994), and they have been found in almost all insect groups (Genta et al., 2003, 2007, 2009; Erthal et al., 2007; Pauchet et al., 2009; Bragatto et al., 2010; Lucena et al., 2011; Moraes et al., 2012; Souza et al., 2016; Souza, 2018).

In addition to their digestive activity, some β-1,3-glucanases, such as those found in the lepidopteran *Helicoverpa armigera* and different termite species, are involved in insect immunity (Bulmer and Crozier, 2006; Pauchet et al., 2009). Insects express several pattern recognition receptors like β-glucan recognition proteins (βGRPs), β-glucan binding proteins (GBP), and Gram-negative bacteria binding proteins (GNBP), involved in the activation of the innate immune response (Ochiai and Ashida, 1988, 2000). These receptors are homologs to some β-1,3-glucanases, but without catalytic activity (Bragatto et al., 2010; Hughes, 2012). Both insect β-1,3-glucanases and β-glucan-binding proteins structurally belong to family 16 of glycoside hydrolases (GHF16) (Genta et al., 2009; Bragatto et al., 2010).

Our current understanding of the intestinal physiology of mosquito larvae is highly incomplete. This issue becomes more relevant when we consider the great potential of larval stages as targets for vector control. In this study, we identified coding sequences for GHF16 in the genome of *Ae. aegypti*, compared the expression of these GHF16 genes in larval tissues and evaluated the physiological role of some GHF16 by knockdown experiments. We showed that β-1,3-glucanases are likely to be involved in digestion and recognition of invading microorganisms in *Ae. aegypti* larvae. Additionally, we were able to identify the gene that expresses the major larval gut β-1,3-glucanase. This enzyme might be an interesting target for inhibition studies and the development of a new generation of larvicides, as β-1,3-glucanases are absent in humans and seem to be essential for mosquito larval physiology.

## Materials and Methods

### Insects Rearing and Maintenance

*Aedes aegypti* eggs (Rockefeller strain) were obtained from the colony of the Laboratory of Physiology and Control or Arthropod Vectors (LAFICAVE/IOC-FIOCRUZ; Dr. José Bento Pereira Lima). Insects were reared until adult stage at 26 ± 2°C and 70 ± 10% relative humidity with a 12-h light/12-h dark cycle. Hatching was induced by adding 100 mL of distilled water into 200 mL plastic cups containing eggs, and then incubating at 28°C for 30 min. After incubation, groups of first instar larvae (*n* = 80) were transferred to plastic bowls containing 100 mL of dechlorinated water and kept at 26 ± 1°C until adult stage. Insects were fed 0.1 g cat food, (Whiskas^®^, Purina, Brazil) following the protocol by Souza et al. (2016). Cat food is one of the standard diets for *Ae. aegypti* larval rearing. This diet has been successfully used by our group as well as by others (Costa et al., 2010; Price et al., 2015). Larvae fed on cat food exhibit expected developmental rates and it also helps reducing laboratory costs. Food was added only once at the beginning of each experiment.

### Identification of GHF16 Sequences in the Genome of *Ae. aegypti*

Genes belonging to the glycoside hydrolase family 16 (GHF16) (defined as described in the CAZy database) in *Ae. aegypti* genome were characterized using FAT software (Seabra-Junior et al., 2011), which integrates HMMER^1^ and BLAST+ tools (Camacho et al., 2009) to filter the initial dataset and perform automatic annotation. The filter step used the HMG-box conserved domain (Pfam code PF00722) to identify and extract only proteins containing such a domain in the *Ae. aegypti* dataset (VectorBase^2^, *Ae. aegypti* Liverpool, AaegL1.3). The annotation step compared the filtered proteins for similarity with proteins and conserved domains databases using BLAST with nr and Swiss-Prot uniprot databases. All results were manually verified.

### Bioinformatic and Phylogenetic Analysis of GHF16 Sequences

Alignment of the homologous sequences coding for GHF16 proteins from *Ae. aegypti* and the generation of consensus sequences were performed using the algorithm CLUSTAL^3^ (Higgins, 1994) and the software BIOEDIT^4^ (Hall, 1999). The sequences obtained were analyzed by the algorithms BLAST^5^ (Altschul et al., 1990), signal IP^6^ (Dyrløv Bendtsen et al., 2004), NETOGlyc 4.0^7^ (Julenius et al., 2005), NETNGlyc 1.0^8^, and ProtParam^9^ (Gasteiger et al., 2003). Trees were generated using MEGA5.05 (Tamura et al., 2011). Bootstrap values were set at 1,000 replications.

### RNA Extraction and cDNA Synthesis

One hundred fourth instar larvae were used to obtain total RNA. Fifty whole (non-dissected) larvae were pooled together, and 50 were dissected to obtain pools of the head, digestive tract and rest of the body. Extractions were performed using the TRI^®^ reagent (SIGMA # T9424) according to the manufacturer’s instructions. After extraction, the RNA was quantified using a Nanodrop^®^ (NanoDrop Technologies, Wilmington, DE, United States). RNA samples were treated with DNase (Turbo DNA-free^TM^, Ambion, AM1907), and reverse-transcribed using a commercial kit following the manufacturer’s protocol (Superscript III First-Strand kit, Invitrogen, Cat. no. 18080-051) and using an oligo dT (18) primer (PRODIMOL Technology). After reverse transcription, samples were treated with RNase H and cDNA was quantitated using a Nanodrop. For further analysis, larval and tissue cDNA samples were normalized to a concentration of 50 ng/μL.

### PCR and Semi-Quantitative RT-PCR

For the amplification of DNA fragments corresponding to GH16 sequences, specific oligonucleotides were designed (Supplementary Table S1). PCR reactions were performed with the GoTaq^®^ DNA Polymerase kit (Promega) using the constitutive gene RP49 as a control (Gentile et al., 2005). Each reaction (20 μL) contained buffer 1X, dNTP (0.2 mM), MgCl_2_ (1.5 mM), oligonucleotides (10 μM each), Taq DNA polymerase (0.025 U) and 1 μL of cDNA or genomic DNA (50 ng/μL). Each reaction consisted in a varied number of cycles with intervals of 1 min at 94°C (denaturation), 30 s at 55°C (annealing) and 1.5 min at 72°C (extension). Different numbers of cycles were performed in each experiment, with 40 cycles for the initial experiments, and a range from 24 to 40 cycles for the semi-quantitative determination of relative expressions, and for the confirmation of the silencing of the genes after larval feeding with dsRNA. Three independent PCRs with at least three different biological samples were performed for each condition.

### Electrophoresis and Densitometric Analysis

PCR and RT-PCR products were subjected to agarose gel electrophoresis with a final concentration of 1% (w/v) in TBE buffer. After electrophoresis, the material was evidenced with ethidium bromide solution (0.5 μg/mL) and visualized in a UV light transilluminator (312 nm). Gels were photographed (E-Gel Image, Life Technologies, United States) and analyzed with the ImageJ program (Sheffield, 2007), generating semi-quantitative profiles of gene expression based on the intensity of bands developed with UV light, subtracting the background from each lane.

### Preparation of dsRNA

Specific primers were designed for the synthesis of dsRNA (Supplementary Table S2). We used the QIAquick PCR Purification Kit or QIAquick Gel Extraction Kit (QIAGEN, United States) to purify PCR products. For the *in vitro* transcription and purification of dsRNA, we used the MEGAscript^®^ RNAi Kit (Ambion, Life Technologies, United States). Purified dsRNA was quantified using Nanodrop^®^.

### Feeding With dsRNA and Measurement of Biological Parameters

We investigated if feeding larvae of *Ae. aegypti* with dsRNA coding for different GHF16 gene sequences could have an impact in the development of fourth instar larvae. In all experiments, larvae in the L2 larval stage were immersed in solutions containing 0.5 μg/μL dsRNA (prepared as above) plus 2.5% (w/v) bromophenol blue according to Singh et al. (2013). Sixty insects were kept in this solution for 2 h at room temperature. After this, 20 insects were selected according to the intensity of the blue color in their digestive tract. Those with gut contents with intense blue color were then transferred to individual pots and maintained until adulthood. After dsRNA treatment, survival and pupation were followed in experiments where 20 larvae were kept in containers containing 100 mL of filtered water and 0.1 g of cat food (Whiskas^®^ – Masterfoods Brasil Alimentos Ltda.). In experiments under stress conditions, in each group after dsRNA treatment, 120 larvae were kept in microtubes containing 1 mL of filtered water and no nutritional source during 2 days. Larval and pupal mortality, pupation and emergence were monitored and recorded daily. Pupation and emergence data were plotted and compared using the Log-rank (Mantel-Cox) test. Mortality and weights were expressed as means ± SEM, and non-transformed data were compared by ANOVA or pairwise *t*-tests. Three biological replicates were performed for each experimental condition.

### Preparation of Samples for Enzymatic Assays

Fifty larvae were immobilized by placing them on the ice, after which they were dissected in cold 0.9% (w/v) NaCl. During dissection of each larva, we separated the head and the entire gut, and the remaining tissues were assembled and named as “rest of body” samples. Ten heads and rest of bodies were homogenized in MilliQ water with the aid of a microtube pestle (Model Z 35, 997-1, Sigma, United States), using the proportion of 100 μL of water for 10 insects. Ten guts were homogenized in 100 μL of cold MilliQ water containing 2.5 μL of 20 mM phenylmethylsulfonyl fluoride (PMSF), 20 μM Pepstatin A and 20 μM *trans*-epoxysuccinyl-L-leucyl amino (4-guanidino)butane (E-64). All samples were centrifuged for 10 min at 14,000 × *g* at 4°C. Both pellets and soluble fractions were stored at -20°C until used as enzyme source for enzymatic assays.

### Enzymatic Assays and Effect of pH

We determined β-1,3-glucanase activity in *Ae. aegypti* larvae by measuring the release of reducing groups from 0.25% (w/v) laminarin from *Laminaria digitata*, (SIGMA Cat. no. L9634) in a thermocycler with a modified bicinchoninic acid reagent (Lucena et al., 2013). The influence of pH in the enzymatic activity was studied using the buffers: sodium citrate (pH 3–7, 200 mM), EPPS (pH 7–9, 200 mM), AMPSO (pH 9–10, 200 mM) and CAPS (pH 10–11, 200 mM) with overlapping pH values to rule out possible inhibition by the buffering species. All assays used the buffers at a final concentration of 83 mM and were performed at 30°C under conditions such that activity was proportional to protein concentration and time. Controls without enzyme or substrate were included. One unit of enzyme (U) is defined as the amount that hydrolyzes one μmol of glycosidic bonds per min, using a glucose standard curve in the same conditions. Comparisons between means of two independent groups were made with a pairwise *t*-test. Results are expressed as the group mean ± SEM.

### Chromatography and Determination of Molecular Masses by Gel Filtration

Samples containing 50 guts, 50 heads, and 50 rest of bodies were homogenized in 100 μL of 10 mM phenylthiourea (PTU) and 600 μL of 50 mM citrate buffer pH 7.0 containing 150 mM NaCl. Additionally, gut samples also were homogenized in 10 μL of PMSF, (20 μM), 10 μL of pepstatin A (20 μM) and 10 μL of E-64 (20 μM). The samples were then centrifuged for 10 min at 10,000 × *g* at 4°C and the soluble fractions were collected. Samples with 500 μL from each of the soluble fractions obtained from tissues were applied into an HR 10/10 Superdex 200 column (GE Healthcare Biosciences) equilibrated with 50 mM citrate buffer pH 7.0 containing 150 mM NaCl. Proteins were eluted with the same buffer (30 mL), with a flow of 0.5 mL/min, and fractions of 0.5 mL were collected and assayed for enzymatic activity. The assays were performed with 35 μL of each fraction and 25 μL of laminarin 0.25% (w/v) in deionized water (Millipore, United States). Molecular mass standards used were: aprotinin (6.5 kDa), cytochrome C (12.4 kDa), bovine serum albumin (66 kDa), alcohol dehydrogenase (150 kDa), amylase (200 kDa), and blue dextran (2,000 kDa). Molecular masses of eluted activities were calculated using the correlation between K_av_ and log_10_ of molecular mass (Bonner et al., 2007).

### Statistical Analysis

Linear regressions were performed using Microsoft Excel (Microsoft). Statistical comparisons were made using GraphPad Prism software (version 7.0, GraphPad Software Inc.). Significance was considered when *p* < 0.05.

## Results

Six sequences of genes coding for proteins belonging to the family 16 of glycoside hydrolases (GHF16) were found in the *Ae. aegypti* genome. They were named AaeGH16.1, AaeGH16.2, AaeGH16.3, AaeGH16.4, AaeGH16.5, and AaeGH16.6 (GenBank codes: EAT44802.1, EAT44801.1, EAT41280.1, EAT40654.2, EAT40654.2, EAT38986.1). These genes contain two to six exons (Supplementary Figure S1).

We performed the alignment of the amino acid sequences of each predicted protein with the homologous sequences found in different databases. We also analyzed aspects such as the presence of peptide signals, glycosylation sites, and conserved catalytic residues. Of the six *Ae. aegypti* GH16 sequences, five (AaeGH16.1, AaeGH16.2, AaeGH16.3, AaeGH16.5, and AaeGH16.6) showed putative signal peptides and the typical conserved catalytic glutamate residues of this GH family that are included in the consensus region SGE(I/V)DL(M/L)ES(R/K). The only protein in this group of GHF16 sequences that did not show a putative signal peptide was AaeGH16.4. AaeGH16.4 also do not present the conserved catalytic glutamate residues (Supplementary Figure S2).

These six GHF16 genes encode proteins with distinct predicted molecular masses and isoelectric points, ranging from 39 to 57 kDa, and from 4.9 to 9.8, respectively. They also present a varying number of putative *N*- and *O*-glycosylated residues, ranging from 0 to 5 and 0 to 4, respectively. All sequences have only one conserved GH16 domain (Table 1).

**Table 1:** Summary of the gene sequence characteristics of the glycoside hydrolases of family 16 that are present in *Ae. aegypti* genome.

Gene	GenBank ID	VectorBase ID	Gene size (bp)	Exons	mRNA size (bp)	CDS (bp)	Protein sequence size (aa)
AaeGH16.1	EAT44802.1	AAEL003889	8,476	3	1,257	1,254	418
AaeGH16.2	EAT44801.1	AAEL003894	8,506	4	1,185	1,182	394
AaeGH16.3	EAT41280.1	AAEL007064	1,287	4	1,116	1,113	371
AaeGH16.4	EAT40654.2	AAEL007626	25,148	6	1,512	1,509	503
AaeGH16.5	EAT40654.2	AAEL009176	1,219	2	1,161	1,158	386
AaeGH16.6	EAT38986.1	AAEL009178	1,300	3	1,185	1,182	394

**Signal peptide (putative)**	**Mature protein residues**	**pI**	**MW**	***N*-glycosylation sites (predicted)**	***O*-glycosylation sites (predicted)**	**GH16 domain sequence (CDD)**	

YES	394	5.7	43,847.36 g/mol	N116, N124, N242, N264, N300	ND	82–417	
YES	368	5.0	41,048.47 g/mol	N91, N301	T40, T41, T107, T346	57–393	
YES	347	9.8	39,428.25 g/mol	ND	ND	47–370	
NO	503	7.6	57,407.02 g/mol	ND	ND	1–326	
YES	370	5.6	41,219.57 g/mol	N135	T28, T29, T334	45–385	
YES	371	4.9	41,881.38 g/mol	N238	T342, T351	55–393	

*CDD, Conserved Domain Database; CDS, protein coding sequence; ND, unidentified.*

**FIGURE 1:**
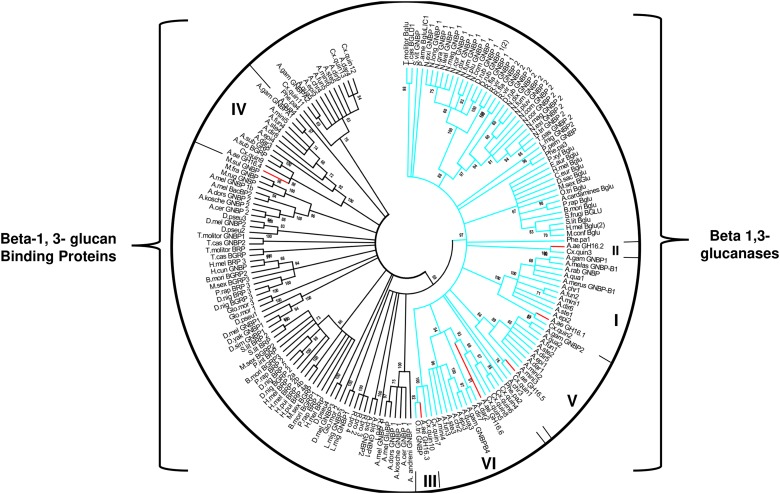
Cladogram of selected protein sequences of insect β-1,3-glucanases, and β-1,3-glucan binding proteins. Branches are statistically supported by bootstrap analysis (cutoff 70%). The blue branches discriminate the monophyletic sequences of the insects that have the conserved catalytic glutamates; the green branches discriminate the paraphyletic group of sequences that do not have the conserved catalytic residues. The bootstrap values were obtained from the analysis of 10,000 replicates, using the Neighbor-Joining algorithm (MEGA software 5.05). Consensus phylogenetic tree used sequences of: *Anopheles gambiae* (AGAP002798-PA, AGAP002799-PA, AGAP002796-PA, AGAP006761-PA, AGAP012409-PA), *Anopheles christyi* (ACHR004102-RA, ACHR005689, ACHR008721-RA, ACHR001881-RA, ACHR009179-RA), *Anopheles darlingi* (ADAR007290-PA, ADAR007286-PA, ADAR006526-PA, ADAR009199-PA), *Anopheles dirus* (ADIR003516-RA, ADIR010616-RA, ADIR003518-RA, ADIR003625-RA, ADIR000553-RA) *Anopheles epiroticus* (AEPI010194-RA, AEPI009256-RA, AEPI005496-RA, AEPI002293-RA), *Anopheles funestus* (AFUN006014-RA, AFUN009437-RA, AFUN006016-RA, AFUN002755-RA, AFUN004083-RA), *Anopheles minimus* (AMIN004837-RA, AMIN003902-RA, AMIN003903-RA, AMIN003900-RA, AMIN010081-RA, AMIN008919-RA), *Anopheles quadriannulatus* (AQUA008516-RA, AQUA009400-RA, AQUA009402-RA, AQUA003848-RA, AQUA014348-RA), *Anopheles stephensi* (ASTE003966-RA, ASTE009324-RA, ASTE009326-RA, ASTE010371-RA, ASTE004573-RA), *Culex quinquefasciatus* (XM_001845911.1, XM_001845228.1, XM_001845913.1, XM_001845759.1, JF907421.1, XM_002135149.1, XM_001845915.1, XM_001847484.1, XM_001847484.1, XM_001845910.1, XM_001845757.1, XM_001864211.1, XM_001845229.1), *Phlebotomus papatasi* (PPATMP000880-PA, PPATMP002587-PA, PPATMP002588-PA, PPATMP010440-PA), *Rhodnius prolixus* (RPRC011769-PA, RPRC003210-PA, ABU96697.1), *Simulium vittatum* (EU930267.1), *Anopheles arabiensis* (ACN38171.1, CAO83421.1), *Anopheles bwambae* (ABU80038.1), *Anopheles melas* (ABU80011.1), *Anopheles merus* (ABU80005.1, AAZ08489.1, AAZ08502.1), *Ochlerotatus triseriatus* (ACU30929.1), *Phlebotomus perniciosus* (ADH94599.1). The code of the other sequences can be seen in Supplementary Table S3.

We also constructed phylogenetic trees using a Neighbor-Joining algorithm to have a better understanding of the relationships among the GH16 genes from *Ae. aegypti* and other insect sequences of this protein family (Figure 1). A second aim was to locate these proteins in two well-known functional groups in GHF16, namely β-glucanases and β-glucan binding proteins. Analysis of GH16 sequences from several insect orders revealed two major clades: one clade includes sequences that lack the catalytic glutamates and another clade including sequences containing the conserved catalytic residues (Figure 1). The same analysis indicates a monophyletic group containing all putative β-1,3-glucanases that bear the conserved catalytic residues. Some of these sequences were annotated in the databases as Gram-Negative Binding Proteins (GNBPs) or glucan binding/recognition proteins (GBPs or GRPs), as is the case of many putative enzymes of the genus *Anopheles*. The cladogram also shows a paraphyletic group of putative β-glucan binding protein sequences lacking the catalytic residues. The arrangement of the sequences in the clades also suggests the occurrence of an expansion in the genes of β-1,3-glucanases in the Nematocera dipterans. Sequences from *Ae. aegypti* containing the catalytic residues (AaeGH16.1, 2, 3, 5, and 6) grouped in branches with sequences of other Nematoceran dipterans, suggesting the diversification of these genes in the ancestor of the suborder.

**FIGURE 2:**
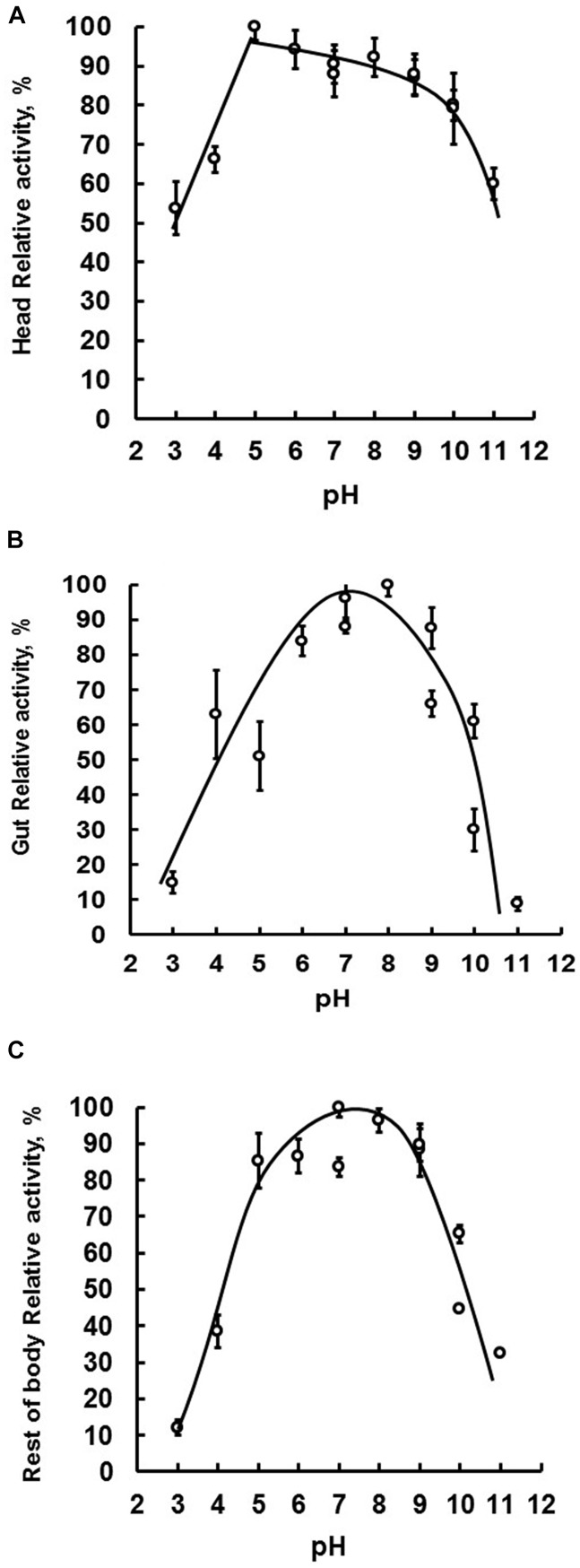
The tissue-specific optimal enzymatic activity of β-1,3-glucanase. Samples were assayed using laminarin as substrate. Optimal activity was determined by performing enzymatic reactions under a pH range of 3–11. Evaluated tissues were head **(A)**, digestive tract **(B)**, and rest of the body **(C)** of fourth instar larval *Ae. aegypti*.

Because several members of GHF16 have β-1,3-glucanase activity, and because previous work has demonstrated this activity in the head, gut and rest of body of *Ae. aegypti* larvae (Souza et al., 2016), we decided to characterize these β-1,3-glucanase activities further. We performed this by estimating and comparing the optimal β-1,3-glucanase activity of these tissues, carrying out enzymatic assays in a range of pH conditions. Tissue-specific *Ae. aegypti* β-1,3-glucanases extracted from the head, gut or rest of body presented maximum activities between pH 6–9, 5–9, and 5–10, respectively (Figure 2).

**FIGURE 3:**
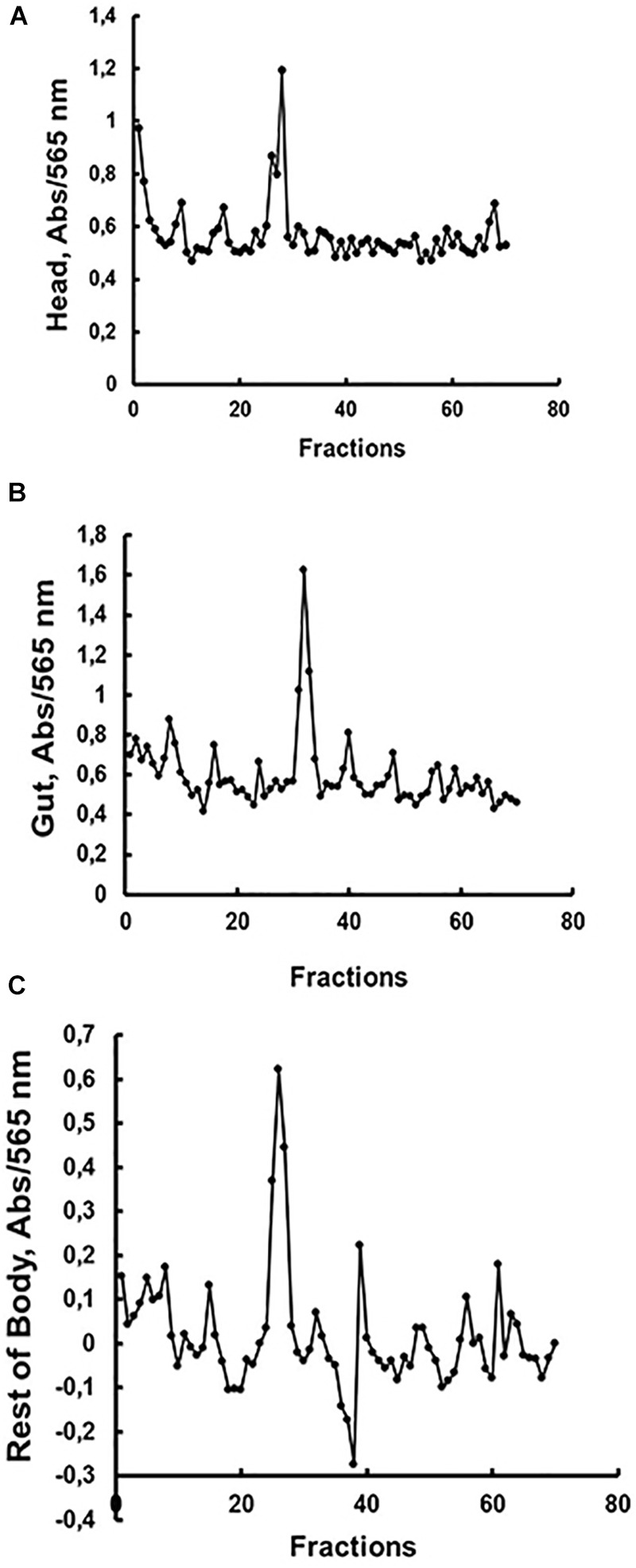
Activity against laminarin in fractions obtained after gel filtration chromatography (Superdex 200 – AKTA FPLC) of soluble fractions obtained from the **(A)** head, **(B)** digestive tract and **(C)** rest of the body of *Ae. aegypti* larvae. Elution volumes and molecular masses of protein standards were used to build a calibration curve for calculation of the molecular masses of β-1,3-glucanase activities. Elution volumes (mL)/fraction (number) of the standards were as follows: 18.22/34 (cytochrome C, 12.4 kDa), 16.86/32 (carbonic anhydrase, 29 kDa), 14.87/28 (bovine serum albumin, 66 kDa), 13.49/25 (alcohol dehydrogenase, 150 kDa), 12.77/24 (β-amylase, 200 kDa), and 5.64/9 (blue dextran, 2000 kDa). The elutions (mL/fraction number) of β-1,3-glucanase activities from **(A)** head, **(B)** gut, and **(C)** rest of body were 13.75/26, 16.75/32, and 13.5/25, respectively, resulting in predicted molecular masses of 142, 41, and 150 kDa.

We decided to submit the soluble fractions from the larval gut, head or rest of body to gel filtration chromatography, in order to compare the presence and molecular masses of β-1,3-glucanase isoforms in these tissues. The results are presented in Figure 3. The β-1,3-glucanase activities from all tissues of *Ae. aegypti* larvae were eluted as one single peak (Figure 3), but with different molecular masses when we compare the tissues to each other. Molecular masses observed for β-1,3-glucanases from head, gut and rest of body were, respectively, 142, 41, and 150 kDa (Figure 3).

We decided to follow the expression of GHF16 coding transcripts in the head, gut, and the rest of the body of *Ae. aegypti* larvae, trying to correlate the expression of some particular gene to the β-1,3-glucanase activities characterized above. Using specific oligonucleotides (Supplementary Table S1), we were able to amplify fragments with the expected size both from genomic DNA (PCR, Supplementary Figure S3) and cDNA (RT-PCR, Supplementary Figure S4). Amplification from genomic PCR confirmed the presence and structure of the six GHF16 genes in the genome of *Ae. aegypti*. Amplification from cDNA obtained from entire larvae (Supplementary Figure S4A), heads (Supplementary Figure S4B), guts (Supplementary Figure S4C) and rest of bodies (Supplementary Figure S4D) showed that all larval tissues express the six GHF16 genes at different levels.

**FIGURE 4:**
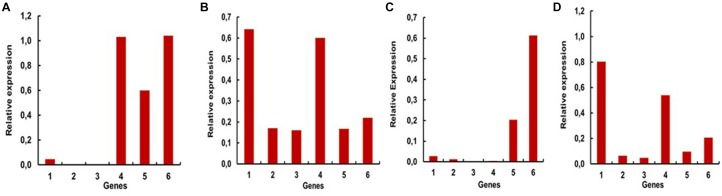
Relative expressions of the genes encoding GHF16 proteins in fourth instar larvae of *Ae. aegypti*, measured in series of semi-quantitative RT-PCR reactions with 24 or 27 cycles. Numbers are relative expressions normalized using the gene RP49 as a constitutive control. cDNA samples were prepared as described in “Materials and Methods” section from **(A)** whole L4 larvae (27 cycles), **(B)** heads (27 cycles), **(C)** guts (24 cycles) and **(D)** rest of bodies (27 cycles). 1–6 correspond to the relative expression levels of AaeGH16.1-6, respectively.

To have a more defined picture of the specificity of the expression levels of these genes in different tissues and the whole larvae, we performed RT-PCR reactions using increasing numbers of cycles for each gene and sample. In this way we obtained saturation curves of the amplified products, allowing us to locate higher levels of expression of one or more genes to the larval stage or specific tissues of the larvae (Figure 4A–D). Whole larvae seem to have higher expression of AaeGH16.4, AeGH16.5, and AaeGH16.6. This is particularly visible after 27 cycles of RT-PCR amplification (Figure 4A). In the head of the larvae, the most expressed genes are AeGH16.1 and AaeGH16.4, a clear picture visible after 27 cycles (Figure 4B). In the gut, AaeGH16.5 and AeGH16.6 are the most expressed genes. This is observed after 24 cycles of amplification (Figure 4C). In the rest of the body, AaeGH16.1 and AeGH16.4 are the most expressed genes (27 cycles; Figure 4D). The genes AaeGH16.2 and AaeGH16.3 appear to have low expression levels in *Ae. aegypti* larvae in general.

**FIGURE 5:**
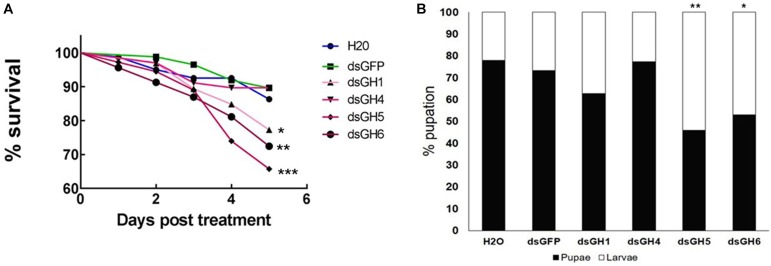
*Ae. aegypti* GHF16 genes have distinct physiological roles. **(A)** Survival curve of *Ae. aegypti* fourth instar larvae after feeding with dsRNA. dsGH1, dsGH4, dsGH5, dsGH6 correspond to larvae fed with dsRNA coding, respectively, for the genes AaeGH16.1, AaeGH16.4, AaeGH16.5, and AaeGH16.6. GFP and H_2_O correspond to the control groups fed with dsGFP or water only. **(B)** Percentage of *Ae. aegypti* fourth instar larvae and pupae in after treatment with dsRNA and following for 12 days in control diet (cat food). GH1/GH4/GH5/GH6/GFP/H_2_O correspond to larvae exposed to dsRNA coding for AaeGH16.1, AaeGH16.4, AaeGH16.5, AaeGH16.6, GFP, and water, respectively. Comparison of survival curves vs. the control treated with dsGFP used the Log-rank test (^∗^*p* < 0.05; ^∗∗^*p* < 0.01; ^∗∗∗^*p* < 0.001) and comparison of groups vs. the control treated with dsGFP used the chi-square test (^∗^*p* < 0.05; ^∗∗^*p* < 0.01).

To have a better understanding of the physiological role of the genes with higher expression in the larval tissues (AaeGH16.1, AaeGH16.4, AaeGH16.5, and AaeGH16.6), we fed third instar larvae of *Ae. aegypti* with dsRNA specific for each gene, checking for RNAi knock-down effects and their resulting phenotypes. As controls, we used larvae fed only with water and larvae fed with dsRNA coding a GFP sequence. We evaluated the survival of the larvae until the fifth day after ingestion of dsRNA. Survival curve data indicate that knockout of the gene AeGH16.5 is associated with increased mortality, followed by AeGH16.6, AeGH16.4, and AeGH16.1 genes, compared to the GFP and water control groups (Figure 5A).

The next parameter evaluated after treatment with dsRNA was pupation of larvae. The group treated with dsRNA coding for AaeGH16.4 was the only one that presented pupation similar to the controls (Figure 5B). Contrastingly, all other treated groups showed lower pupation rates, especially the larvae treated with dsRNA coding for the gene AeGH16.5, which had the lowest proportion of pupae produced during the experiment (Figure 5B). No changes were observed in the external appearance or general behavior of the larvae in any of the experimental groups.

**FIGURE 6:**
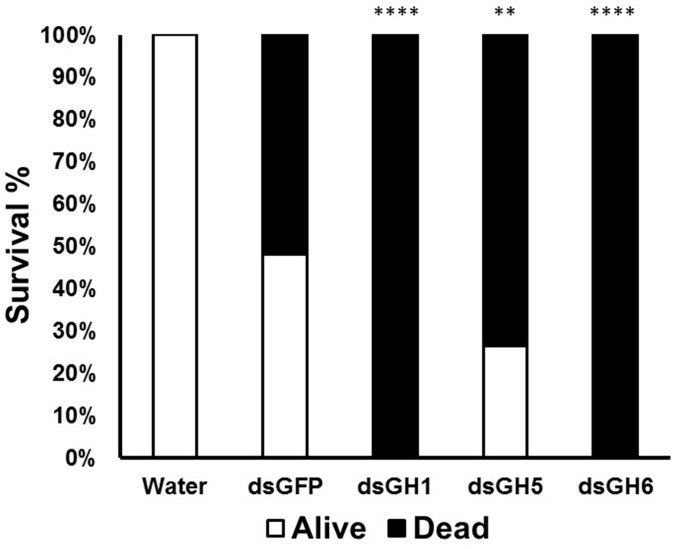
Mortality and survival of *Ae. aegypti* fourth instar larvae treated with dsRNA under conditions of high density and food restriction. GH1, GH5, and GH6 correspond to insects treated with dsRNA coding for AaeGH16.1, AaeGH16.5, and AaeGH16.6, respectively. GFP and H_2_O correspond to controls treated with dsRNA coding for GFP and water, respectively. Comparison of groups *vs* the control treated with dsGFP used the Fisher’s exact test (^∗∗^*p* < 0.01; ^∗∗∗∗^*p* < 0.0001).

Mosquito larvae go through enormous biotic and abiotic challenges that affect their development and survival. Once we detected moderate phenotypes both in larval survival (Figure 5A) and pupation (Figure 5B), we decided to repeat these experiments under stress conditions. We monitored larvae after the treatment of larvae with dsRNA coding for genes whose knockdown resulted in higher mortality in the larvae and a longer pupation delay (AeGH16.1, AeGH16.5, and AeGH16.6). We isolated a larger number of larvae and restricted them in much smaller compartments and, after treatment with dsRNA, no food was added for 2 days. These unfavorable conditions of high density and food restriction resulted in the death of all larvae after treatment with dsRNA coding for the genes AaeGH16.1 and AaeGH16.6 (Figure 6). Larvae treated with water only showed 100% survival, and groups treated with dsRNA for GFP or AaeGH16.5 showed intermediate survival proportions, around 30–50% (Figure 6).

**FIGURE 7:**
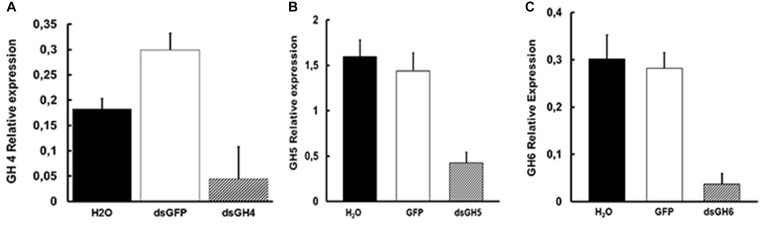
Levels of expression of the genes AeGH16.4 (GH4), AeGH16.5 (GH5), and AeGH16.6 (GH6) in *Ae. aegypti* fourth instar larvae after treatment with dsRNA. **(A)** Relative expression of the gene AeGH16.4 in whole larvae. **(B)** Relative expression of the gene AeGH16.5 in the gut. **(C)** Relative expression of the gene AeGH16.6 in the rest of the body. Expression levels were normalized using the ribosomal RP gene 49 as the constitutive marker. We used 24 cycles for AeGH16.4 and 27 cycles for AeGH16.5 and AeGH16.6 in the RT-PCR reactions. See “Materials and Methods” for details.

Despite the observation of phenotypes described above, we decided to confirm the knockdown effect of genes AeGH16.1, AeGH16.4, AeGH16.5, and AeGH16.6 after feeding larvae with dsRNA. For that, 5 days after treatment, we isolated the total RNA from whole larvae, gut and rest of bodies, and analyzed the expression levels of each transcript. We were able to observe a significant silencing effect on whole larvae for the AeGH16.4 gene, in the digestive tract only for the AeGH16.5 gene and in the rest of the body only in the AeGH16.6 gene (Figure 7). The relative expression levels of AeGH16.5 and AeGH16.6 in the rest of body and gut, respectively, were not changed after treatment with their correspondent dsRNA (data not shown).

**FIGURE 8:**
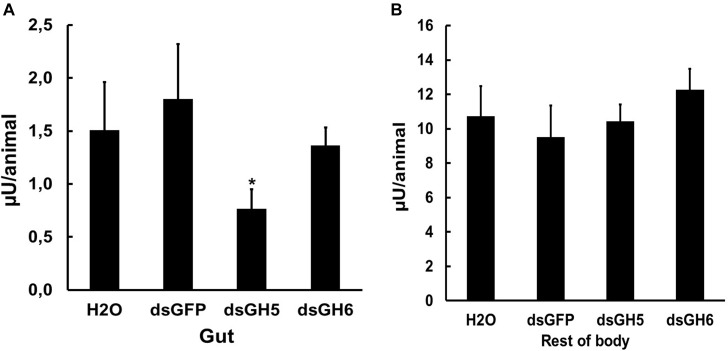
β-1,3-glucanase activity (laminarin as substrate) in the soluble fractions of the digestive tract **(A)** and rest of the body **(B)** of fourth instar larvae of *Ae. aegypti* treated with water (H_2_O) or dsRNA coding for GFP, AaeGH16.5 (dsGH5), and AaeGH16.6 (dsGH6). Comparison of groups *vs.* the control treated with dsGFP used the *t*-test (^∗^*p* < 0.05).

To further investigate the physiological and biochemical role of GHF16 genes in *Ae. aegypti*, we decided to assay the β-1,3-glucanase activity in larvae silenced for the genes AaeGH16.5 and AaeGH16.6. Several insect GHF16 genes are digestive β-1,3-glucanases, and AaeGH16.5 and AaeGH16.6 showed their highest relative expression in the gut of larvae (Figure 4). We observed a significant decrease in the β-1,3-glucanase activity in the gut soluble fraction of larvae treated with dsRNA coding for AeGH16.5 gene, with no changes in the gut activity of larvae treated with dsRNA coding for AaeGH16.6 or GFP (Figure 8A) when compared to controls treated with water only. No changes in the soluble β-1,3-glucanase activity of the rest of the body were observed after any of the treatments above (Figure 8B).

**FIGURE 9:**
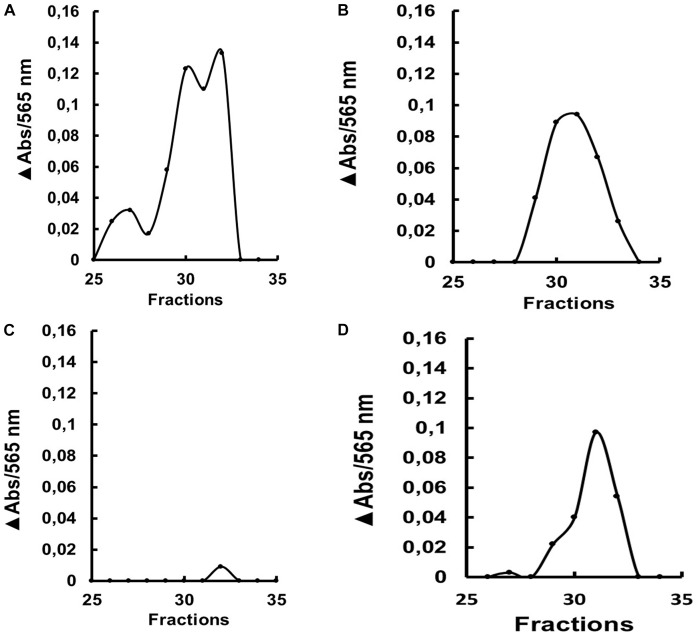
β-1,3-glucanase activity (laminarin substrate) after gel filtration chromatography (Superdex 200 column/AKTA-FPLC) of the soluble fraction of the gut from L4 larvae of *Ae. aegypti* after ingestion of **(A)** water, **(B)** dsRNA coding for GFP, **(C)** dsRNA coding for AaeGH16.5 and **(D)** dsRNA coding for AaeGH16.6. The data presented is the absorbance increase (ΔAbs) relative to the baseline of the chromatographic profile after incubation with laminarin. The experiment was performed independently twice.

We submitted the soluble fractions of the gut from larvae treated with dsRNA to gel filtration chromatographies, to verify if some change in the molecular mass of the major isoform had occurred after treatment. Gut samples from the insects fed with dsRNA coding for AeGH16.5 showed a significantly lower activity peak in the chromatographic profile when compared to the other groups (Figure 9).

## Discussion

Intestinal β-1,3-glucanases were already described in cockroaches (Genta et al., 2003), termites (Lucena et al., 2011), grasshoppers (Genta et al., 2007), beetles (Genta et al., 2009), moth larvae (Pauchet et al., 2009; Bragatto et al., 2010), sandfly larvae (Moraes et al., 2012, 2014; Vale et al., 2012), and recently in *Ae. aegypti* larvae (Souza et al., 2016). These enzymes belong to the family 16 of glycoside hydrolases (Davies and Henrissat, 1995; Coutinho et al., 1999). The search in the genome of *Ae. aegypti* revealed six sequences with shared characteristics among members of the family 16 of glycoside hydrolases (Table 1 and Supplementary Figure S1), such as the PFAM-00722 domain (Bateman et al., 2004). Five of the six *Ae. aegypti* sequences (AeGH16.1, AeGH16.2, AeGH16.3, AeGH16.5, and AeGH16.6) have conserved glutamate residues within the consensus region SGE(I/V)DL(M/L)ES(R/K), acting as donors and proton acceptors for the enzymatic activity of β-1,3-glucanases (Hahn et al., 1995), while such residues were absent in the AeGH16.4 sequence (Supplementary Figure S2).

The active site glutamate residues are essential for the catalytic activity of β-1,3-glucanases (Zhang et al., 2003), and are conserved in all glucanases subfamilies but not in β-1,3-glucan binding proteins (βLP) or β-1,3-glucan recognizing proteins (βRP). Thus, it is likely that *Ae. aegypti* has five β-1,3-glucanases with conserved catalytic regions and that AeGH16.4 would be a βLP or βRP, having no signal peptide sequence or conserved catalytic residues. The functional divergence between glucanases and βLP/βRP is known to be involved in the loss of the catalytic activity of β-1,3-glucanase and the addition of an N-terminal region or a carbohydrate recognition domain (Zhang et al., 2003; Pauchet et al., 2009). Since they are homologous proteins, some authors suggest that β-1,3-glucanases and βLP/βRP originated from the duplication of an ancestral β-1,3-glucanase gene originating in the predecessor of Hexapoda, and that βLP/βRP had lost their catalytic activities but maintained the characteristic of recognition and binding to polysaccharides such as β-1,3-glucans (Bragatto et al., 2010; Hughes, 2012).

β-1,3-glucanases and β-glucan binding proteins belonging to GHF16 seem to be evolutionary related (Pauchet et al., 2009; Bragatto et al., 2010; Hughes, 2012). Consistently, two main clades were found in our analysis (Figure 1). (1) A monophyletic clade assembling the sequences with conserved catalytic residues (β-1-3-glucanases) and (2) a paraphyletic clade, which did not show catalytic residues (βLP). It has been proposed that the animal β-1,3 glucanase ancestral gene suffer a duplication. Thus, insects should bear at least two copies of genes from GHF16 (Bragatto et al., 2010). Our results show that many additional Nematoceran dipteran sequences are clustered into monophyletic sub-branches of β-1,3-glucanases, suggesting that several duplication events might have occurred in the β-1,3-glucanase gene family, resulting in at least five β-glucanase paralog genes in the genomes of *Culex*, *Aedes,* and *Anopheles*.

The presence of several GHF16 sequences in the genome of *Ae. aegypti*, *C. quinquefasciatus,* and *A. gambiae* strongly suggests that this gene family suffered duplication and diversification during the evolutionary establishment of the Culicidae ancestor. That gene expansion might be related to the adaptation of the larvae for the aquatic environment, with a higher exposition to pathogens. However, it is not clear how catalytically active β-1,3-glucanases might participate in the insect immune response, as the canonical function described for GHF16 proteins in the immune cascade is the recognition of pathogen-associated molecular patterns (PAMPs). The presence of conserved catalytical residues and enzymatic activity contrast with a binding role, as glucanases tend to dissociate from their substrates after the hydrolytic cleavage of glycosidic bonds. In some cases, a processive mode of action and a secondary non-catalytical binding site have been described, resulting in a more stable association with the recognized polysaccharide (Genta et al., 2007).

The optimum pH of *Ae. aegypti* larvae β-1,3-glucanases is similar to the observed in other insects as *Periplaneta americana* (pHo = 6; Genta et al., 2003), *Tenebrio molitor* (pHo = 6; Genta et al., 2009), *Abracris flavolineata* (pHo = 6; Genta et al., 2007), and *Lutzomyia longipalpis* (pHo = 6–8; Moraes et al., 2012). Although the maximum activity range of the enzymes in the different tissues is similar, the comparison suggests the presence of different β-1,3-glucanases among the tissues of the larvae (Figure 2). Coherently, β-1,3-glucanases obtained from each tissue presented different molecular masses (Figure 3). The molecular mass of β-1,3-glucanases recovered from head and rest of body were strikingly different from the observed for the enzyme from the gut, which showed a molecular mass similar to the measured for another insect intestinal β-1,3-glucanases (Genta et al., 2003, 2007, 2009; Bragatto et al., 2010).

The luminal pH of *Ae. aegypti* midgut is buffered around 7, 11, 8, and 7 in the gastric caeca, anterior midgut, posterior midgut, and hindgut, respectively (Linser et al., 2009). It is interesting to notice that gut and head β-1,3-glucanases of this insect retain significant activities even at highly basic pH values like 10, with 80 and 50% of maximal activity, respectively. In this way, these enzymes are probably active in the anterior compartments of the midgut, where initial digestion of the ingested fungal cells might take place. A recent report suggests that ingested yeast cells are killed quickly, probably in the initial portions of the larval midgut (Souza et al., 2016).

Beside the biochemical characterization, new approaches as molecular biology studies are required to understand better the role of β-1,3-glucanases in *Ae. aegypti* larvae. All six GHF16 genes were expressed at different levels in larvae (Supplementary Figure S4). To date, gene expression studies in *Ae. aegypti* larvae are scarce. In general, they show a high specificity for particular stages in the expression of developmental genes (Harker et al., 2013).

Experiments using the RNAi technology have greatly enhanced knowledge about gene functions and, because of their specificity, the RNAi technique also offers excellent potential for pest control strategies (Baum et al., 2007; Price and Gatehouse, 2008; Huvenne and Smagghe, 2010; Diaz-Albiter et al., 2011; Singh et al., 2013). Silencing of AeGH16.5 resulted in the highest observed mortality rate, followed by AaGH16.6, AaGH16.1, and AaGH16.4 (Figure 5A). Accordingly, AaeGH16.5 knockdown resulted in the lowest pupation rate, followed by AaeGH16.6 (Figure 5B). Mortality and the smaller pupation rate in the larvae fed with the dsRNA targeting the AeGH16.5 gene suggest a digestive function since the development of the larvae seems to be impaired.

It has been shown that nutritional stress in mosquito larvae leads to the emergence of adults with immune deficiency (Telang et al., 2012). To test a possible phenotype related to the immune function of these genes, we kept the larvae under stress conditions for 5 days after gene silencing. The larvae were kept in small containers with high population density and were not fed for 2 days. We observed that these conditions resulted in 100% mortality in larvae knocked-down for the genes AeGH16.1 and AeGH16.6. The AaeGH16.5 knockdown also resulted in high mortality, but at similar rates than the dsGFP control (Figure 6). It is possible that the genes AeGH16.1 and AeGH16.6 exert an immune function in the larvae and that the adverse conditions in which the larvae remained during 5 days have challenged this system, culminating in their death.

We were able to obtain transcriptional knock-down results in whole larvae for the AeGH16.4 gene, and in gut and rest of the body for the genes AaeGH16.5 and AaeGH16.6, respectively (Figure 7). Although these two last genes are more expressed in the gut, AeGH16.6 has been knocked-down only in the rest of the body. The reason for this tissue specificity in silencing is still not clear, but this lack of response in some tissues must be considered when interpreting the phenotypes for each gene.

It was not possible to obtain an effective knock-down of the gene AeGH16.1. One of the generally limiting factors for the RNAi technique is the internalization of the dsRNA by cells. Techniques such as soaking, feeding or injection of dsRNA require the absorption of dsRNA molecules by the cells, which may or may not occur. In this work, we believe that the knock-down effect due to ingestion of dsRNA can spread from the gut to other tissues of the body, as described in other studies (Zhang, 2010; Singh et al., 2013). Considering that, it is also not clear to us why the gene AaeGH16.1 was not silenced in the conditions tested. It is noteworthy that even without a significative knockdown we were able to detect important phenotypes in the larvae treated with dsRNA targeting this gene. It is possible that even a small or temporary knockdown, not detected by semi-quantitative RT-PCR, resulted in physiological impairment, resulting in larval mortality (Figure 5A), delay in pupation (Figure 5B) and inability to cope with nutritional stress (Figure 6).

The knockdown experiments of all the GHF16 genes tested here resulted in moderate phenotype under normal conditions and severe phenotype under stressful conditions. In insects, most of the RNAi experiments using the soaking technique were performed using cell lines. The soaking technique appears to present a less potent knockdown than microinjections directly into the hemocoel, due to barriers such as the insect cuticle. Thus, it is possible that the observed knockdown for some GHF16 genes in *Ae. aegypti* larvae has not reached its full potential. Nevertheless, our experiments strongly suggest that it is possible to use the soaking technique for functional screenings on whole insects efficiently.

β-1,3-glucanase enzymatic assays were also performed on tissue samples obtained from silenced *Ae. aegypti* larvae. Gut and rest of the body samples were evaluated in larvae silenced for the genes AaeGH16.5 and AeGH16.6. In the assays of guts of larvae treated with dsRNA for AaeGH16.5, the β-1,3-glucanase activity suffered a considerable decrease; however, there were no changes in the activity found in the rest of the body (Figure 8). Gel filtration chromatography showed a significantly lower peak of β-1,3-glucanase for the gut of larvae treated with dsRNA for AeGH16.5 when compared to larvae treated with dsRNA for AeGH16.6 or the control groups (Figure 9). These data strongly suggest that AeGH16.5 code for the major digestive β-1,3-glucanase of *Ae. aegypti* larvae. Due to the small size of *Ae. aegypti* larvae and small amounts of protein in gut samples from this insect, the identification of digestive enzymes using the traditional techniques of purification and characterization are strongly hindered. In this way, the use of an efficient knockdown strategy was extremely important for the identification of the major gut β-1,3-glucanase of this insect as coded by the gene AaeGH16.5.

One putative role of the AaeGH16.5 protein in the gut of *Ae. aegypti* larvae is the digestion of ingested fungal cells. The dependence on β-1,3-glucanase activity for the breakdown of yeast cells in *Ae. aegypti* was already established using lytic assays and competition with laminarin, a canonical substrate for this enzyme (Souza et al., 2016). In other insects, gut β-1,3-glucanases were already implicated in digestion of fungal cells (Genta et al., 2003, 2009; Lucena et al., 2011; Moraes et al., 2012, 2014) or plant hemicelluloses (Genta et al., 2007; Bragatto et al., 2010). In some insects, the gut β-1,3-glucanase activity was correlated to the innate immune defense against pathogens at the mucosa level (Bulmer et al., 2009; Pauchet et al., 2009). Despite that, when we consider the complexity of the diet of *Ae. aegypti* larvae, it is not possible to discard a role in the digestion of plant hemicelluloses, as the relative contribution of ingested plant or fungal cells is unknown. Independently of the substrate that is recognized by this enzyme, it is clear from the phenotypes of larval death and pupation arrest, observed after knockdown of AaeGH16.5, that the nutrient acquisition which results from its action is critical for larvae development and pupation.

Regarding the other genes studied, it is noteworthy that the properties of the amino acid sequences coded by AaeGH16.1, AaeGH16.2, AaeGH16.3, and AeGH16.6 suggest that these proteins are secreted β-1,3-glucanases, having catalytic activity and being probably secreted to the extracellular space. These sequences contain the conserved catalytic glutamates which are essential for the hydrolytic mechanism of GHF16, and a putative signal peptide, suggesting secretion via the canonical exocytic route. Interestingly, AaeGH16.6 is also expressed preferentially in the gut of larvae, in a pattern that resembles AaeGH16.5. Additionally, knockdown of AaeGH16.6 resulted in larval mortality and arrest of pupation. However, no change in the total β-1,3-glucanase activity in the gut or the body was observed after treatment with dsRNA targeting this gene, and the chromatographic profile of secreted intestinal β-1,3-glucanase activity was not changed either. It is possible that AaeGH16.6 codes for an enzyme that is insoluble, unstable, associated with a different tissue than the gut (e.g., hemocytes), or acts against other substrates than laminarin. Activities against the most varied substrates, like agar, carrageenan, xyloglucan or hyaluronic acid, have been described in GHF16, totalizing 14 different activities (Lombard et al., 2014). From these, only two, endo-1,3-β-glucanase (EC 3.2.1.39) and endo-1,3(4)-β-glucanase (EC 3.2.1.6) would show significant activity against laminarin. In this respect, the activity of the protein coded by AaeGH16.6 and its exact role in the gut physiology of the larvae needs further investigation.

The genes AaeGH16.2 and AaeGH16.3 are not preferentially expressed in the larval stage when compared to the other GHF16 of *Ae. aegypti*. It is possible that these genes exert their role in the pupae or adults, and investigation about the presence of β-1,3-glucanases activity in these stages might clarify their role.

The data gathered here also suggest an immune role for the gene AaeGH16.1. This gene is expressed preferentially in the head and rest of body of larvae, and its sequence codes for the conserved catalytic glutamates and a putative signal peptide. Silencing of AaeGH16.1 resulted in moderate larval mortality, pupation arrest, and pronounced mortality in stress conditions. β-1,3-glucanases were already reported for the head and rest of body of *Ae. aegypti* larvae (Souza et al., 2016), so it is possible that AaeGH16.1 is the gene responsible for the expression of these activities. Interestingly, the molecular masses observed for β-1,3-glucanases in samples from the tissues above exceed the predicted molecular mass of AaeGH16.1 in 100 kDa, suggesting that in this case the AaeGH16.1 protein might be associated to other proteins from the innate immune cascade, as serine proteases or prophenoloxidases. Binding to other proteins in a macromolecular complex is a standard feature of insect β-1,3-glucan binding proteins (Cerenius et al., 2010; Baxter et al., 2017).

The gene AaeGH16.4 seems to code a β-glucan binding protein associated with the innate immune cascade. Its function may be inferred from the lack of the conserved catalytic residues of GHF16 and the lack a putative signal peptide. The expression pattern of AaeGH16.4 also suggests a systemic role not related to the digestive system, as it is preferentially expressed in the head and rest of body of larvae. Besides that, knockdown of AaeGH16.4 resulted in no detectable phenotype in control conditions, suggesting that this protein is involved in the immune response and not in nutrient acquisition. However, further experiments need to be performed to prove this hypothesis, especially using challenges with recognized pathogens and more biochemical assays.

The description of the β-1,3-glucanase activity in Culicidae larvae is recent (Souza et al., 2016; Souza, 2018). β-1,3-glucanase might be an essential enzyme for the digestion of *Ae. aegypti* larvae, and its absence in mammals make this activity an interesting target for inhibition (CAZY^10^). β-1,3-glucanase might be an important factor for the digestion of fungi in mosquito larvae, because the disruption of cells by mouthparts in insects is negligible, and the breakdown of polysaccharides in the cell wall by enzymes is necessary to release nutrients as proteins, glycogen and nucleic acids (Terra and Ferreira, 1994, 2005). In this respect, we may consider the possibility to explore GHF16 proteins as targets for inhibition and control of mosquito larvae. Proteinaceous inhibitors of β-1,3-glucanases have been described in marine algae (Ermakova et al., 2001). Besides that, GHF16 proteins are absent in mammals (Lombard et al., 2014), indicating that inhibitors of β-1,3-glucanases might not have a binding target in humans, with consequent low toxicity. Inhibition of insect β-1,3-glucanases has been explored with drastic effects in survival (Bulmer et al., 2009). However, more detailed studies about the structure, specificity, and function of *Ae. aegypti* GHF16 proteins are necessary for the development and validation of this strategy. The study of this protein family in mosquitoes may reveal new aspects of insect–pathogen relationships and help the development of new targets for the control of insect vectors.

## Conclusion

The genome of *Ae. aegypti* has six genes encoding GHF16 proteins. Comparative sequence analysis, gene expression, and functional studies of these genes allowed us to identify AaeGH16.5 as the gene coding for the major gut β-1,3-glucanase in the larvae of *Ae. aegypti*. Besides that, AaeGH16.1, AaeGH16.4, and AaeGH16.6 seem to be related to the innate immune response. These findings may improve our understanding of physiology and evolution of Culicidae larvae, as well as potentiate the development of new strategies for vector control.

## Author Contributions

RSS, HD-A, RS, JL, and MG performed the experiments and analyzed the data. RSS and FG conceived the study and wrote the manuscript. All authors read and approved the final manuscript.

## Conflict of Interest Statement

The authors declare that the research was conducted in the absence of any commercial or financial relationships that could be construed as a potential conflict of interest.

## Footnotes

1 http://hmmer.janelia.org/

2 http://www.vectorbase.org

3 http://www.ch.embnet.org/software/ClustalW.html

4 http://www.mbio.ncsu.edu/BioEdit/bioedit.html

5 http://blast.ncbi.nlm.nih.gov/Blast.cgi

6 http://www.cbs.dtu.dk/services/SignalP/

7 http://www.cbs.dtu.dk/services/NetOGlyc/

8 http://www.cbs.dtu.dk/services/NetNGlyc/

9 http://web.expasy.org/protparam/

10 www.cazy.org

## Supplementary Material

FIGURE S1 Schematic diagram of exons and introns in the genes coding for glycoside hydrolases of family 16 in the *Ae. aegypti* genome.

FIGURE S2 Alignment (Clustal X program) of the amino acid sequences of members of the family 16 of glycoside hydrolases in the *Ae. aegypti* genome. Hypothetical glycosylation sites are in bold and underlined, residues with similar properties are in gray and fully conserved residues in black. Putative signal peptides are boxed. The catalytic glutamate residues are marked by a circle.

FIGURE S3 PCR amplification of fragments of genes encoding GHF16 proteins from the genomic DNA of *Ae. aegypti*. Lane 1 – 1 kb molecular standard PROMEGA # 100787-018; Lane 2 – RP49 – ribosomal constitutive gene; Lanes 3–8: AeGH16.1, AeGH16.2, AeGH16.3, AeGH16.4, AeGH16.5, and AeGH16.6, respectively. Genomic DNA was extracted from larvae, and specific primers were used for each gene. After 40 cycles of amplification, the PCR products were analyzed on agarose gel and developed with ethidium bromide.

FIGURE S4 Amplification of fragments of the transcripts encoding GHF16 proteins from the cDNA obtained from **(A)** Entire fourth instar larvae, **(B)** heads, **(C)** digestive tracts, and **(D)** rest of bodies of *Ae. aegypti* larvae. Lane 1 – Molecular standard of 100 bp PROMEGA # 15628-019; Lanes 2–7 – AeGH16.1, AeGH16.2, AeGH16.3, AeGH16.4, AeGH16.5, and AeGH16.6, respectively. Lane 8 – RP49 – constitutive ribosomal gene. RNA was extracted from the larvae and tissues, the cDNA generated from that RNA by RT was used for PCR reactions with specific primers. After 40 cycles of amplification, the PCR products were analyzed on agarose gel and developed with ethidium bromide.
