# Supplementary figures and images for: Biochemical and Functional Characterization of Glycoside Hydrolase Family 16 Genes in Aedes aegypti Larvae: Identification of the Major Digestive β-1,3-Glucanase
Source: Front Physiol. 2019 Feb 28;10:122. doi: 10.3389/fphys.2019.00122 (PMC6403176; doi:10.3389/fphys.2019.00122)

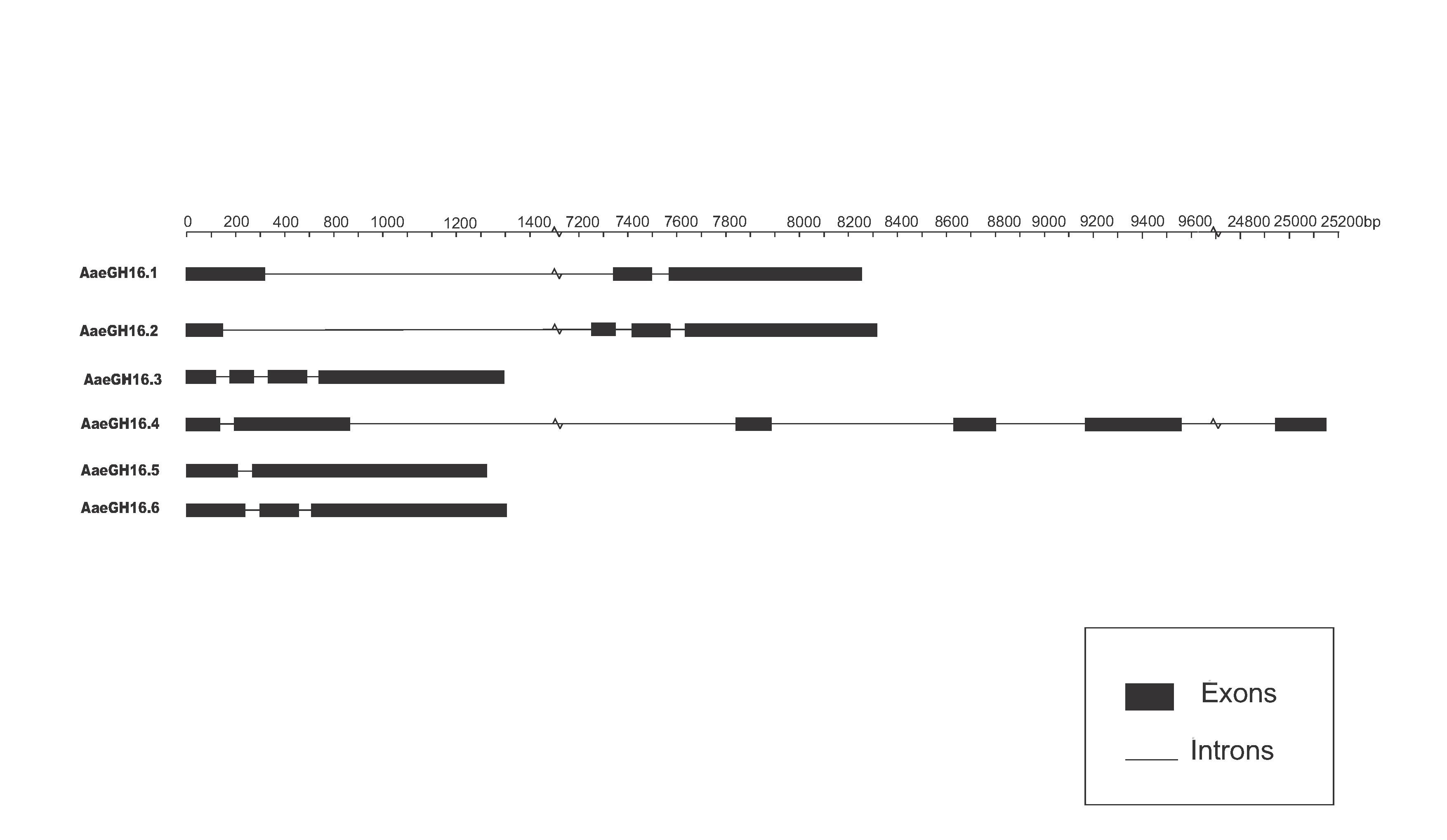

Supplement: FIGURE S1 — Schematic diagram of exons and introns in the genes coding for glycoside hydrolases of family 16 in the Ae. aegypti genome. [file Image_1.JPEG]

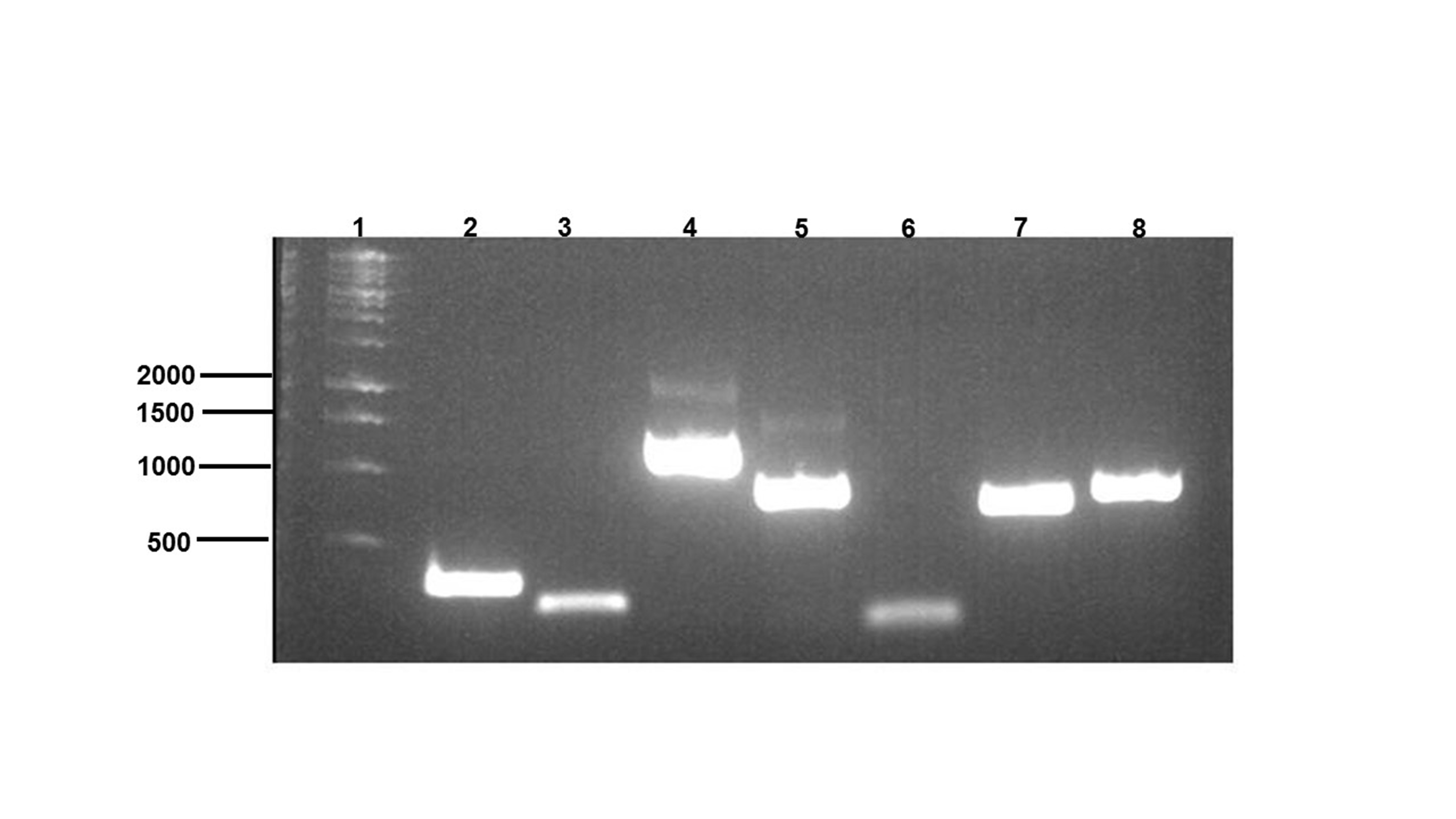

Supplement: FIGURE S3 — PCR amplification of fragments of genes encoding GHF16 proteins from the genomic DNA of Ae. aegypti. Lane 1 – 1 kb molecular standard PROMEGA # 100787-018; Lane 2 – RP49 – ribosomal constitutive gene; Lanes 3–8: AeGH16.1, AeGH16.2, AeGH16.3, AeGH16.4, AeGH16.5, and AeGH16.6, respectively. Genomic DNA was extracted from larvae, and specific primers were used for each gene. After 40 cycles of amplification, the PCR products were analyzed on agarose gel and developed with ethidium bromide. [file Image_3.JPEG]

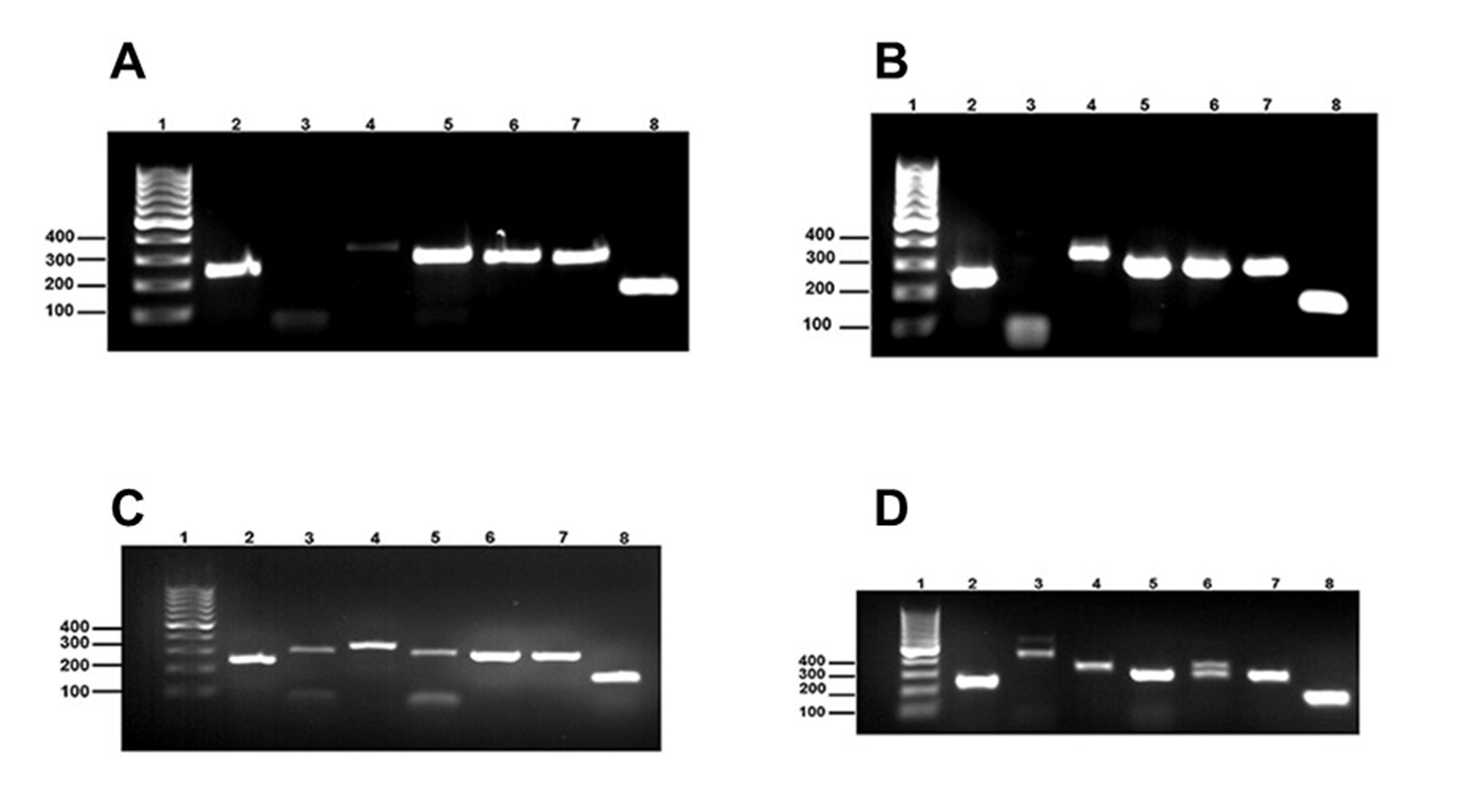

Supplement: FIGURE S4 — Amplification of fragments of the transcripts encoding GHF16 proteins from the cDNA obtained from (A) Entire fourth instar larvae, (B) heads, (C) digestive tracts, and (D) rest of bodies of Ae. aegypti larvae. Lane 1 – Molecular standard of 100 bp PROMEGA # 15628-019; Lanes 2–7 – AeGH16.1, AeGH16.2, AeGH16.3, AeGH16.4, AeGH16.5, and AeGH16.6, respectively. Lane 8 – RP49 – constitutive ribosomal gene. RNA was extracted from the larvae and tissues, the cDNA generated from that RNA by RT was used for PCR reactions with specific primers. After 40 cycles of amplification, the PCR products were analyzed on agarose gel and developed with ethidium bromide. [file Image_4.jpg]
