# Supplementary material for: Biochemical and Functional Characterization of Glycoside Hydrolase Family 16 Genes in Aedes aegypti Larvae: Identification of the Major Digestive β-1,3-Glucanase
Source: Front Physiol. 2019 Feb 28;10:122. doi: 10.3389/fphys.2019.00122 (PMC6403176; doi:10.3389/fphys.2019.00122)
Supplement: Supplementary file 5 [file Table_1.DOCX]

Table S1. Sequences of oligonucleotide primers used for semi-quantitative RT-PCR of transcripts encoding the GHF16 family in *A.aegypti* larvae. F: sense primer (Foward). R: antisense primer (Reverse).

| **Gene** | **Sequence** |
| --- | --- |
| **AaeGH16.1F** | GTGACACTGAACCGAGATCG |
| **AaeGH16.1R** | TGGGACGAATGTAAAGGGTG |
| **AaeGH16.2F** | GCATGAAAACACTCTGGCTG |
| **AaeGH16.2R** | TCAAATCTCGGTTTCCCTTCG |
| **AaeGH16.3F** | TCTATACATCCGCCCAACATTG |
| **AaeGH16.3R** | CCATCAGATCCACTTCACCTG |
| **AaeGH16.4F** | GGATTCCAGTATGCTACCCTG |
| **AaeGH16.4R** | ACGTAATCCACTTTCAGCTCC |
| **AaeGH16.5F** | GAGGAAAGTGCCCCGAAG |
| **AaeGH16.5R** | AGGTTCAAAGTTCCACTGCTC |
| **AaeGH16.6F** | GACTTTCTGCTCTGGTGATTTG |
| **AaeGH16.6R** | TCTTTCACAACCATCCCACG |

Table S2. Sequences of oligonucleotide primers used to amplify the cDNA fragments used in the preparation of dsRNA.

| **Gene** | **Sequence** |
| --- | --- |
| **dsGH16.1F** | TAATACGACTCACTATAGGGAGAACCCGTCAGTTTGGGCAGACGC |
| **dsGH16.1R** | TAATACGACTCACTATAGGGAGAGACGTTGAACCCCTTGCTGTATTC |
| **dsGH16.4F** | TAATACGACTCACTATAGGGAGAATGGGCATCGGTTGCGAGGTGG |
| **dsGH16.4R** | TAATACGACTCACTATAGGGAGAGTATGAAAGTTATCGCTGAAATGCTC |
| **dsGH16.5F** | TAATACGACTCACTATAGGGAGAGTGTTGAACAGGTTGGTTCAACTTT |
| **dsGH16.5R** | TAATACGACTCACTATAGGGAGAAAATGGAAGCCGTTGTTGAAACCTT |
| **dsGH16.6F** | TAATACGACTCACTATAGGGAGAGCGTTGAGCAAGTAGGATCGACTT |
| **dsGH16.6R** | TAATACGACTCACTATAGGGAGACGATGGAAGTCATTGTTGAAGCCT |

Table S3. Sequences used in the consensus tree. The sequences were obtained from GenBank or Vector Base (in italic).

| **Organism** | **Code** | **Organism** | **Gene ID** |
| --- | --- | --- | --- |
| *Acyrthosiphon pisum* | XM_001947795 | *Anopheles christyi* | *ACHR004102* |
| *Acyrthosiphon pisum* | XM_001944438 | *Anopheles christyi* | [*ACHR005689*](https://www.vectorbase.org/Anopheles_christyi/Gene/Summary?db=core;g=ACHR005689;r=KB693113:5974-7379;t=ACHR005689-RA) |
| *Anopheles gambiae* | *AGAP002798* | *Anophele schristyi* | *ACHR008721* |
| *Anopheles gambiae* | *AGAP002799* | *Anopheles christyi* | *ACHR001881* |
| *Anopheles gambiae* | *AGAP002796* | *Anopheles christyi* | *ACHR009179* |
| *Anopheles gambiae* | *AGAP004456* | *Anopheles darlingi* | *ADAR007290* |
| *Anopheles gambiae* | *AGAP006761* | *Anopheles darlingi* | *ADAR007286* |
| *Anopheles gambiae* | *AGAP012409* | *Anopheles darlingi* | *ADAR006526* |
| *Anopheles gambiae* | *AGAP004455* | *Anopheles darlingi* | *ADAR009199* |
| *Armigeres subalbatus* | AAT99011 | *Anopheles dirus* | *ADIR003516* |
| *Drepanotermes rubriceps* | AAZ08491 | *Anopheles dirus* | *ADIR010616* |
| *Drepanotermes rubriceps* | AAZ08504 | *Anopheles dirus* | *ADIR003518* |
| *Drosophila melanogaster* | AF228472 | *Anopheles dirus* | *ADIR003625* |
| *Drosophila melanogaster* | AF228474 | *Anopheles dirus* | *ADIR000553* |
| *Drosophila melanogaster* | AF228473 | *Anopheles epiroticus* | *AEPI009258* |
| *Glossina morsitans* | ABC25063 | *Anopheles epiroticus* | *AEPI010194* |
| *Tribolim castenaum* | LOC660764 | *Anopheles epiroticus* | *AEPI009256* |
| *Delias nigrina* | ACI32830 | *Anopheles epiroticus* | *AEPI005496* |
| *Delias nigrina* | ACI32829 | *Anopheles epiroticus* | *AEPI002293* |
| *Euphydryas aurinia* | AEV66277 | *Anopheles funestus* | *AFUN006014* |
| *Locusta migratoria* | AFD54025 | *Anopheles funestus* | *AFUN009437* |
| *Locusta migratoria* | AFD54026 | *Anopheles funestus* | *AFUN006016* |
| *Locusta migratoria* | AFD54027 | *Anopheles funestus* | *AFUN002755* |
| *Manduca sexta* | AAN10151 | *Anopheles funestus* | *AFUN004083* |
| *Manduca sexta* | ADK39022 | *Anopheles gambiae* | *AGAP002798* |
| *Manduca sexta* | AEV66276 | *Anopheles gambiae* | *AGAP002799* |
| *Anopheles arabiensis* | ACN38171 | *Anopheles gambiae* | *AGAP004456* |
| *Anopheles arabiensis* | CAO83421 | *Anopheles gambiae* | *AGAP002796* |
| *Anopheles bwambae* | ABU80038 | *Anopheles gambiae* | *AGAP004455* |
| *Anopheles gambiae* | ABU80032 | *Anopheles gambiae* | *AGAP006761* |
| *Anopheles gambiae* | ACN38130 | *Anopheles gambiae* | *AGAP012409* |
| *Anopheles gambiae* | CAO83469 | *Anopheles minimus* | *AMIN004837* |
| *Anopheles melas* | ABU80011 | *Anopheles minimus* | *AMIN003902* |
| *Anopheles merus* | ABU80005 | *Anopheles minimus* | *AMIN003903* |
| *Anopheles quadriannulatus* | ACN38184 | *Anopheles minimus* | *AMIN003900* |
| *Anthocharis cardamines* | ACI32832 | *Anopheles minimus* | *AMIN010081* |
| *Apis andreniformis* | ACT66878 | *Anopheles minimus* | *AMIN008919* |
| *Apis andreniformis* | ACT66880 | *Anopheles quadri* | *AQUA008516* |
| *Apis cerana* | ACT66877 | *Anopheles quadri* | *AQUA009400* |
| *Apis cerana* | ACT66883 | *Anopheles quadri* | *AQUA009402* |
| *Apis dorsata* | ACT66875 | *Anopheles quadri* | *AQUA003848* |
| *Apis dorsata* | ACT66881 | *Anopheles quadri* | *AQUA014348* |
| *Apis koschevnikovi* | ACT66879 | *Anopheles stephensi* | *ASTE003966* |
| *Apis koschevnikovi* | ACT66882 | *Anopheles stephensi* | *ASTE009324* |
| *Apis mellifera* | ACT66876 | *Anopheles stephensi* | *ASTE009326* |
| *Apis mellifera* | ACT66884 | *Anopheles stephensi* | *ASTE010371* |
| *Armigeres subalbatus* | AAT99011 | *Anopheles stephensi* | *ASTE004573* |
| *Bombyx mori* | ACU57045 | *Culex quinquefasciatus* | *CPIJ004321* |
| *Colias eurytheme* | ACI32831 | *Culex quinquefasciatus* | *CPIJ003613* |
| *Culex quinquefasciatus* | AEQ27734 | *Culex quinquefasciatus* | *CPIJ003612* |
| *Culex quinquefasciatus* | AEQ27734 | *Culex quinquefasciatus* | *CPIJ004320* |
| *Delias nigrina* | ACI32829 | *Culex quinquefasciatus* | *CPIJ004324* |
| *Delias nigrina* | ACI32830 | *Culex quinquefasciatus* | *CPIJ004323* |
| *Diatraea saccharalis* | ABR28479 | *Culex quinquefasciatus* | *CPIJ004231* |
| *Drepanotermes rubriceps* | AAZ08504 | *Culex quinquefasciatus* | *CPIJ004325* |
| *Drepanotermes rubriceps* | AAZ08491 | *Culex quinquefasciatus* | *CPIJ008997* |
| *Drosophila melanogaster* | AAF33849 | *Culex quinquefasciatus* | *CPIJ004229* |
| *Drosophila melanogaster* | AAF33850 | *Culex quinquefasciatus* | *CPIJ013557* |
| *Drosophila melanogaster* | AAF33851 | *Culex quinquefasciatus* | *CPIJ013556* |
| *Drosophila simulans* | AAQ64712 | *Culex quinquefasciatus* | *CPIJ005217* |
| *Drosophila yakuba* | AAQ65045 | *Glossina morsitans* | *GMOY011181* |
| *Euphydryas aurinia* | AEV66277 | *Glossina morsitans* | *GMOY010453* |
| *Galleria mellonella* | CAK22401 | *Glossina morsitans* | *GMOY011180* |
| *Glossina morsitans* | ABC25063 | *Phlebotomus papatasi* | *PPATMP000880* |
| *Heliconius melpomene* | AEV66278 | *Phlebotomus papatasi* | *PPATMP002587* |
| *Helicoverpa armigera* | ABU98621 | *Phlebotomus papatasi* | *PPATMP002588* |
| *Helicoverpa armigera* | ACI32825 | *Phlebotomus papatasi* | *PPATMP010440* |
| *Helicoverpa armigera* | ACI32826 | *Rhodnius prolixus* | *RPRC011769* |
| *Helicoverpa armigera* | ACI32827 | *Rhodnius prolixus* | *RPRC003210* |
| *Helicoverpa armigera* | ACI32828 | *Spodoptera littoralis* | ACH56895 |
| *Hepialus pui* | ADZ45540 | *Spodoptera littoralis* | ACI32819 |
| *Hepialus pui* | ADZ45541 | *Spodoptera frugirpeda* | ABR28478 |
| *Mamestra configurata* | AEA76308 | *Spodoptera litura* | AEQ33590 |
| *Mamestra configurata* | AEA76308 | *Tenebrio molitor* | ACS36221 |
| *Manduca sexta* | AEV66276 | *Tenebrio molitor* | BAC99308 |
| *Manduca sexta* | AAF44011 | *Tenebrio molitor* | BAG14263 |
| *Manduca sexta* | AAN10151 | *Tumulitermes pastinator* | AAZ08503 |
| *Manduca sexta* | ADK39022 | *Apis mellifera* | XM_001121634 |
| *Manica rubida* | ACT66840 | *Apis mellifera* | FJ546099 |
| *Myrmica brevispinosa* | ACT66839 | *Apis mellifera* | FJ546107 |
| *Myrmica fracticornis* | ACT66838 | *Armigeres subalbatus* | AY603183 |
| *Myrmica fracticornis* | ACT66838 | *Bombyx mori* | NM_001043375 |
| *Myrmica lobicornis* | ACT66832 | *Bombyx mori* | NM_001135200 |
| *Myrmica rubra* | ACT66835 | *Bombyx mori* | NM_001166142 |
| *Myrmica ruginodis* | ACT66831 | *Bombyx mori* | NM_001043985 |
| *Myrmica rugulosa* | ACT66837 | *Bombyx mori* | NM_001163714 |
| *Myrmica sabuleti* | ACT66836 | *Culex quinquefasciatus* | XM_001845911 |
| *Myrmica scabrinodis* | ACT66833 | *Culex quinquefasciatus* | XM_001845228 |
| *Myrmica sulcinodis* | ACT66834 | *Culex quinquefasciatus* | XM_001845913 |
| *Nasutitermes comatus* | AAZ08480 | *Culex quinquefasciatus* | XM_001845759 |
| *Nasutitermes comatus* | AAZ08493 | *Culex quinquefasciatus* | JF907421 |
| *Nasutitermes corniger* | AEK64800 | *Culex quinquefasciatus* | XM_002135149 |
| *Nasutitermes corniger* | AEK64801 | *Culex quinquefasciatus* | XM_001845915 |
| *Nasutitermes dixoni* | AAZ08481 | *Culex quinquefasciatus* | XM_001847484 |
| *Nasutitermes dixoni* | AAZ08494 | *Culex quinquefasciatus* | XM_001847484 |
| *Nasutitermes exitiosus* | AAZ08482 | *Culex quinquefasciatus* | XM_001845910 |
| *Nasutitermes exitiosus* | AAZ08495 | *Culex quinquefasciatus* | XM_001845757 |
| *Nasutitermes fumigatus* | AAZ08483 | *Culex quinquefasciatus* | XM_001864211 |
| *Nasutitermes fumigatus* | AAZ08496 | *Culex quinquefasciatus* | XM_001845229 |
| *Nasutitermes graveolus* | AAZ08484 | *Drosophila pseudoobscura* | XM_001352735 |
| *Nasutitermes graveolus* | AAZ08497 | *Drosophila pseudoobscura* | XM_001353956 |
| *Nasutitermes longipennis* | AAZ08485 | *Drosophila pseudoobscura* | XM_002135150 |
| *Nasutitermes longipennis* | AAZ08498 | *Drosophila pseudoobscura* | XM_001353957 |
| *Nasutitermes magnus* | AAZ08486 | *Glossina morsitans* | DQ307159 |
| *Nasutitermes magnus* | AAZ08499 | *Simulium vittatum* | EU930267 |
| *Nasutitermes pluvialis* | AAZ08487 | *Spodoptera frugiperda* | EF641300 |
| *Nasutitermes pluvialis* | AAZ08500 | *Pieris rapae* | ACI32821 |
| *Nasutitermes triodiae* | AAZ08488 | *Pieris rapae* | ACI32824 |
| *Nasutitermes triodiae* | AAZ08501 | *Pieris rapae* | ACI32822 |
| *Nasutitermes walkeri* | AAZ08489 | *Pieris rapae* | ACI32823 |
| *Nasutitermes walkeri* | AAZ08502 | *Plodia interpunctella* | AAM95970 |
| *Ochlerotatus triseriatus* | ACU30929 | *Plutella xylostella* | ACI32820 |
| *Ostrinia nubilalis* | ACI32836 | *Reticulitermes flavipes* | ADJ19004 |
| *Periplaneta americana* | ABR28480 | *Reticulitermes flavipes* | AEK64796 |
| *Phlebotomus perniciosus* | ADH94599 | *Reticulitermes virginicus* | AEK64797 |
| *Rhodnius prolixus* | ABU96697 | *Reticulitermes virginicus* | ADJ19023 |
| *Simulium vittatum* | ACH56895 |  |  |
